# Assessment of respiratory mechanics and respiratory muscles of difficult to wean critically ill patients

**DOI:** 10.1186/2197-425X-3-S1-A313

**Published:** 2015-10-01

**Authors:** D Marouli, K Vaporidi, E Kondili, D Georgopoulos, G Prinianakis

**Affiliations:** University Hospital of Heraklion, Intensive Care Medicine Department, Heraklion, Greece

## Introduction

Weaning from mechanical ventilation represents the period of transition from total ventilator support to spontaneous breathing. Patients who are difficult to wean from mechanical ventilation represent a clinical problem which is usually multifactorial.

## Objectives

To analyze parameters of respiratory system mechanics and to assess diaphragmatic function in ventilator-dependent patients, with normal cardiac function, after repeated unsuccessful weaning trials.

## Methods

In this observational study, 20 stable intubated patients with normal cardiac function undergoing prolonged ventilation (>10 days) in whom 3 attempts of spontaneous breathing trials failed, were evaluated. Diaphragmatic muscle function was assessed invasively by the tension-time index of the diaphragm (TTdi), an indicator of diaphragm endurance time. Additional physiologic measurements of respiratory system were obtained using esophageal balloon catheter.

## Results

The frequency to tidal volume ratio (f/Vt), measurement of rapid shallow breathing, was 125.84 ( ± 111.75) breaths/L, whereas 30% of the patients were above the standard “cut off” value of 105 breaths/L. Dynamic lung compliance and pulmonary resistance at midinspiratory volume were 0.09 ( ± 0.07)L/cmH2O and 16.48 ( ± 7.16) cmH2O/L/s respectively. Of the patients studied, 15 (75%) had increased pulmonary resistance (>10cmH2O/L/s) and 20% of the patients had reduced dynamic lung compliance. The dynamic intrinsic positive end-expiratory pressure (PEEPi, dyn) was 2.11 ± 1.58 cmH2O. Additionally, 30% of the patients had decreased respiratory muscle strength, maximum inspiratory pressure (MIP) < -25cmH2O (-37.16 ± 15.06 cmH2O) whereas 50% of the patients presented limited diaphragm endurance time, TTdi > 0.16 (0.13 ± 0.07). The study group increased the Pdiswing/Pdimax ratio (tidal transdiaphragmatic pressure over maximum transdiaphragmatic pressure) (0.46 ± 0.6), in order to compensate the increased load of respiratory system mainly due to elevated pulmonary resistance. The respiratory time fraction (Ti/TTOT) remained normal (0.30 ± 0.11).

## Conclusions

Weaning from mechanical ventilation continues to be an area of significant interest. Difficult - to- wean patients with normal cardiac function, after prolonged mechanical ventilation, are characterized by reduced diaphragmatic endurance and dysfunction and alterations in respiratory mechanics, as characterized mainly by increased airway resistance.

## Grant Acknowledgment

None of the authors has any conflicts of interest.
